# Correction: Chromatin structure, transcriptional activity and DNA repair efficiency affect the outcome of chemotherapy in multiple myeloma

**DOI:** 10.1038/s41416-020-0830-x

**Published:** 2020-04-03

**Authors:** M. Gkotzamanidou, P. P. Sfikakis, S. A. Kyrtopoulos, C. Bamia, M. A. Dimopoulos, V. L. Souliotis

**Affiliations:** 1 Department of Medical Oncology, Jerome Lipper Multiple Myeloma Center, Dana-Farber Cancer Institute, Harvard Medical School, Boston, MA 02115 USA; 2 Department of Clinical Therapeutics, University of Athens School of Medicine, 11528 Athens, Greece; 3 First Department of Propedeutic Medicine, University of Athens School of Medicine, 11527 Athens, Greece; 4 Institute of Biology, Medicinal Chemistry and Biotechnology, National Hellenic Research Foundation, 48 Vassileos Constantinou Avenue, 11635 Athens, Greece; 5 Department of Hygiene, Epidemiology and Medical Statistics, University of Athens School of Medicine, 11527 Athens, Greece

Correction to: *British Journal of Cancer* (2014) **111**, 1293–1304; https://doi.org/10.1038/bjc.2014.410, published online 22 July 2014

Since the publication of this paper, the authors have been alerted by a reader to a duplication of the NTS non-treated and treated bands appearing in Fig. 2a. The authors would like to apologise for this error, which occurred during the compilation of the Figure, but does not alter the conclusions of the paper. The correct version of Fig. 2a is provided here.

**Fig. 2:**
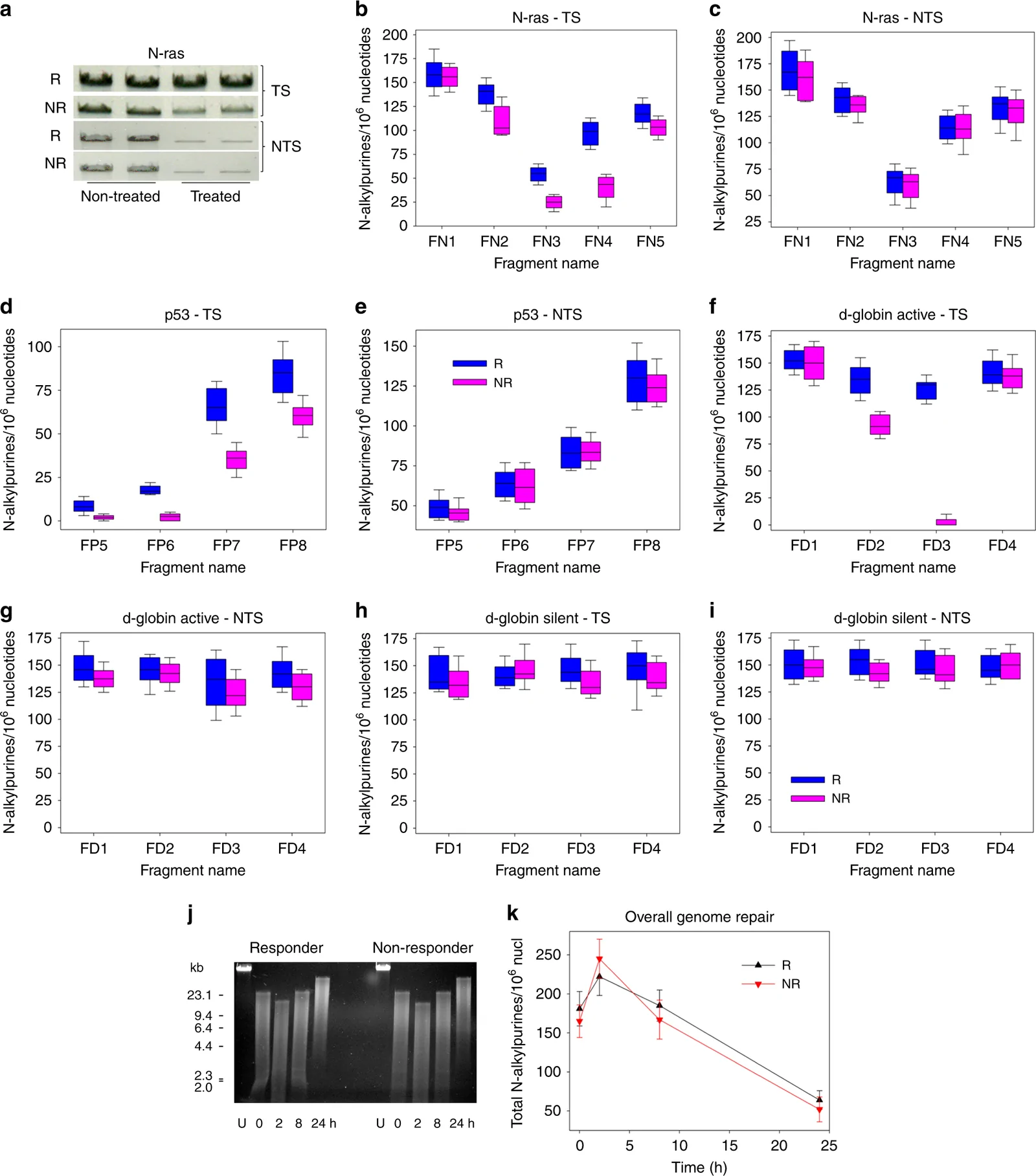
Region-specific repair of melphalan-induced damage along the N-ras, p53and d-globingenes in BMPCs. **a** Representative autoradiograms showing southern blot analysis of melphalan-dependent loss of signal from the full-length (non-treated) band as a result of N-alkylpurine formation in the TS and NTS of the FN4 fragment of the N-rasgene from one MM patient responder (R) and one non-responder (NR) to melphalan therapy. Box plots showing statistical distribution of melphalan-induced N-alkylpurine levels in responders and non-responders to melphalan therapy, in different regions of the TS (**a**, **b**, **d**, **f**, **h**) and the NTS (**a**, **c**, **e**, **g**, **i**) of the active N-ras (**a**–**c**) and p53genes (**d**, **e**) in all patients, the active d-globingene in 11 out of 15 MM patients (**f**, **g**) and the silent d-globingene in 4 out of 15 MM patients (**h**, **i**). The horizontal lines within the boxes represent the median value and the vertical lines extending above and below the boxes indicate maximum and minimum values, respectively. **j** Overall genome repair at various time points (0–24 h) following melphalan treatment of BMPC from one patient responder and non-responder to melphalan therapy. DNAs were electrophoresed in a 0.6% agarose gel and stained with ethidium bromide. Kb kilobases, U untreated samples. **k** Presented are data derived from densitometric analysis of the overall genome repair. The data are based on two biological experiments and several gels from each. The error bars represent s.d. nucl nucleotides.

